# The impact of breast density notification on rescreening rates within a population-based mammographic screening program

**DOI:** 10.1186/s13058-021-01499-4

**Published:** 2022-01-15

**Authors:** Sarah Pirikahu, Helen Lund, Gemma Cadby, Elizabeth Wylie, Jennifer Stone

**Affiliations:** 1 Genetic Epidemiology Group, School of Population and Global Health, The University of Western Australia, 35 Stirling Highway, M431, Crawley, Perth, WA 6009 Australia; 2 BreastScreen Western Australia, Women and Newborn Health Service, Perth, WA Australia; 3 School of Medicine, The University of Western Australia, Perth, WA Australia

**Keywords:** Breast density notification, Rescreening, Mammographic screening, Breast cancer

## Abstract

**Background:**

High participation in mammographic screening is essential for its effectiveness to detect breast cancers early and thereby, improve breast cancer outcomes. Breast density is a strong predictor of breast cancer risk and significantly reduces the sensitivity of mammography to detect the disease. There are increasing mandates for routine breast density notification within mammographic screening programs. It is unknown if breast density notification impacts the likelihood of women returning to screening when next due (i.e. rescreening rates). This study investigates the association between breast density notification and rescreening rates using individual-level data from BreastScreen Western Australia (WA), a population-based mammographic screening program.

**Methods:**

We examined 981,705 screening events from 311,656 women aged 40+ who attended BreastScreen WA between 2008 and 2017. Mixed effect logistic regression was used to investigate the association between rescreening and breast density notification status.

**Results:**

Results were stratified by age (younger, targeted, older) and screening round (first, second, third+). Targeted women screening for the first time were more likely to return to screening if notified as having dense breasts (Percent_unadjusted_ notified vs. not-notified: 57.8% vs. 56.1%; *P*_adjusted_ = 0.016). Younger women were less likely to rescreen if notified, regardless of screening round (all *P* < 0.001). There was no association between notification and rescreening in older women (all *P* > 0.72).

**Conclusions:**

Breast density notification does not deter women in the targeted age range from rescreening but could potentially deter younger women from rescreening. These results suggest that all breast density notification messaging should include information regarding the importance of regular mammographic screening to manage breast cancer risk, particularly for younger women. These results will directly inform BreastScreen programs in Australia as well as other population-based screening providers outside Australia who notify women about breast density or are considering implementing breast density notification.

**Supplementary Information:**

The online version contains supplementary material available at 10.1186/s13058-021-01499-4.

## Introduction

Mammographic breast density, the white radiographic appearance of epithelial and stromal tissue on a mammogram, is a strong and established predictor of breast cancer risk [1]. Mammographic breast density also significantly reduces the sensitivity of mammography to detect the disease [2] as tumours also appear white on a mammogram and are hard to detect within dense breast tissue. There is currently no evidence-based screening recommendations for women with dense breasts and therefore many mammographic screening programs do not routinely record or report breast density to its participants. However, consumer advocates argue that women should be notified if they have dense breasts so they can discuss options, including supplemental screening if appropriate, with their doctors. As a result of consumer advocacy, there are increasing mandates for routine breast density notification in the United States and in Canada, and the Food and Drug Administration is currently working on prescribed language for federal breast density notification.

High participation in mammographic screening is essential for its effectiveness to detect cancers early and thereby, improve breast cancer outcomes. The literature regarding the effects of breast density notification on screening participation is sparse and mostly includes reports comparing summary outcomes before and after notification enactment, with no individual-level data. For example, comparisons of overall mammographic screening participation rates in California pre- and post-notification enactment, showed a decrease for women aged 40–70 (pre 82.4% vs. post 77.2%, *P* < 0.001) [3]. In the state of New Jersey, a decrease in overall mammographic screening was also seen 18 months post enactment (pre-legislation 51.5% vs. post 48.5%) [4]. Overall screening participation rates are known to fluctuate (e.g. due to changing demographics or media coverage), so only individual-level data can definitively determine whether breast density notification affects participation outcomes. There are currently no reports in the literature on whether breast density notification effects the likelihood of whether a woman returns to screening when next due (i.e. rescreening rates).

In Australia, mammographic screening is free for all women aged 40+ via population-based BreastScreen programs and women aged 50–74 are actively targeted for routine biennial screening (50–69 prior to July 1, 2013). The BreastScreen Western Australia (WA) program screens around 125,000 women each year and around 56% of women between the targeted ages of 50–74 years [5]. Age-standardized rescreening rates are ~ 50%, ~ 60%, and ~ 80% for women screened for the first time, second time, and in third/subsequent rounds, respectively [5]. Hence, despite the demonstrated benefits of free mammographic screening, half of women fail to return for routine mammography after their first screen. BreastScreen WA is currently the only state program that notifies women if they have dense breasts and have been doing so for over a decade. It is unknown if breast density notification impacts rescreening rates. Previous work investigating the impact of breast density notification on women’s knowledge, psychosocial response and post-screening action [6–8] within BreastScreen WA demonstrated that ~ 91% of notified women indicated that they intended to rescreen again when next due (similar to that in controls, ~ 92%). However, empirical evidence of association is needed to directly inform state mammographic screening policy as well as provide important evidence to other breast cancer screening providers both nationally and internationally that either notify or are considering notifying women about breast density.

Therefore, using individual-level data from women who attended BreastScreen WA from 2008 to 2017, we examined rescreening rates by breast density notification status. We estimated the odds of rescreening for women who were notified they had dense breasts compared to those who were not notified, stratified by age group and screening round, and adjusted for other potential rescreening predictors.

## Materials and methods

Individual-level information for all women who attended screening from January 2008 to March 2020 was extracted from the BreastScreen WA database (*N* = 1,316,688 screens from *N* = 365,698 women). Individual-level information is self-reported via a one-page questionnaire at the time of screening. For each screening event, information included age, screening round, country of birth, Aboriginal status, family history, English spoken at home status, disability status, postcode and breast density notification status. Screening round refers to the number of times a woman has attended screening and categorized as first screen, second screen and third/subsequent screens. Within this report, the term Aboriginal is used in preference to Aboriginal and Torres Strait Islander, in recognition that Aboriginal people are the original inhabitants of WA. No disrespect is intended to our Torres Strait Islander colleagues and community.

Mammography is free for all women aged 40+ however younger women (aged 40–49) are not actively invited to screen (but are subsequently invited to rescreen). Women in the targeted age group are routinely identified via the electoral roll and invited to screen (and rescreen). The targeted age range was 50–69 years but changed to 50–74 years as of July 1, 2013. The targeted age range used for rescreening (prior to July 1, 2013) was 50–67 rather than 50–69, because women aged 68–69 at the time of their screen would be outside the targeted age range of 50–69 when they would be due for their rescreen. The targeted age range for women screened from July 1, 2013, onwards changed to 50–72 [5]. Older women were/are not invited to rescreen if the next scheduled screen is/was due beyond the age 69 and 74 years, respectively. However, older women can make an appointment to screen/rescreen should they choose without an invitation. The three age groups (younger, targeted, and older) depend on whether a woman was invited to screen (i.e. targeted) and/or invited to rescreen. Depending on screening date, targeted women were aged 50–67 or 50–72 years and older women were either 68+ or 72+ years. Younger and targeted women were invited to rescreen but older women were not.

Family history (FH) of breast cancer was categorized as none, a FH, and a significant FH and based on responses from the one-page questionnaire administered at the time of screening. A significant FH included women with ≥ 2 or more first-degree relatives with breast cancer, or a first-degree relative diagnosed < 50 years of age, or a first-degree relative with bilateral breast cancer, or a personal history of ovarian cancer. A FH includes women with first-degree relatives with breast cancer without meeting the above categories. Women with a significant FH of breast cancer or a personal history of breast or ovarian cancer are recommended annual screening.

Postcode was used to assign women to the Accessibility/Remoteness Index of Australia (ARIA) and deciles of the Index of Socio-Economic Disadvantage of the Socio-Economic Indexes for Areas (SEIFA) [9] via Australian Bureau of Statistics census data. SEIFA was grouped as low (deciles 1–4), medium (deciles 5–6) and high (deciles 7–10), where a high score represents an area with fewer people on lower incomes and in unskilled occupations. ARIA was grouped into those residing in major cities, inner regional, outer regional, remote and very remote areas. SEIFA and ARIA information is not available for postcodes associated with a P.O Box mailing address.

### Breast density measurement

Breast density measurement was dichotomous; a radiologist assessment of a Breast Imaging-Reporting and Data System (BIRADS) category c (heterogeneously dense) or d (extremely dense) is considered dense. For women identified as having dense breasts, the BreastScreen WA routine results letter states that mammograms are significantly less sensitive for diagnosing breast cancer in women with dense breasts and recommends they consult their doctor.

### Definition of rescreening

The primary outcome was whether a woman rescreened or not. Women-recommended biennial screening were classified as having rescreened if they returned to BreastScreen WA within 27 months from a previous screen and 15 months for women-recommended annual screening. Rescreening rates were calculated as the number of women who returned to screening within their 27- or 15-month window, out of the number of women who had the opportunity to rescreen.

### Exclusions

**Fig. 1:**
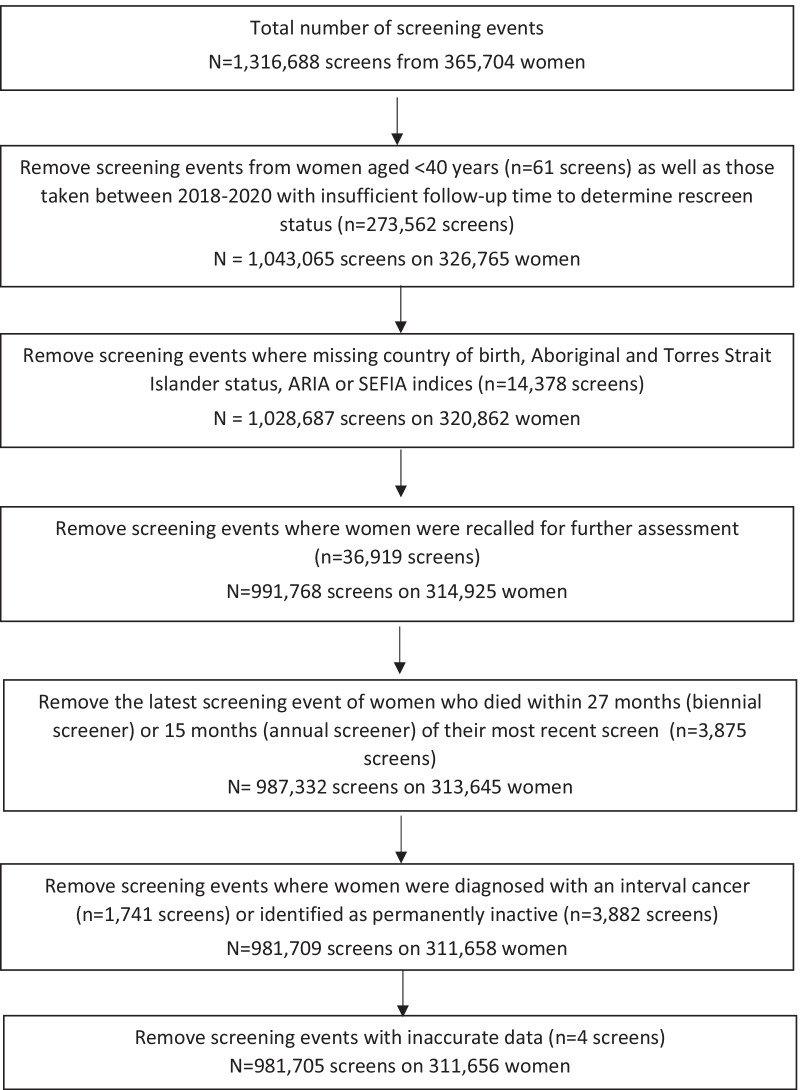
Flowchart of exclusions

Women who were recalled for further assessment were excluded as they were not sent the routine results letter (*n* = 36,919). This includes women who were subsequently diagnosed with breast cancer. Screening events were excluded where women died within the rescreening window (*n* = 4440), and were identified as permanently inactive from screening (*n* = 3882), or aged < 40 years (*n* = 61). Screening events from January 1, 2018 (*n* = 273,562), were excluded as the 27-month rescreening window extended past the study end date. Screening events with missing data were also excluded (*n* = 14,378). A total of 981,705 screening events from 311,656 women were used. A flowchart of exclusions is given in Fig. 1.

### Subset analysis

A subset of screening events from women aged 40–49 who did not rescreen within the recommended screening interval (*n* = 46,420 screening events from 40,780 women) was created to investigate whether notifying women in their 40’s was associated with the likelihood of returning to screening at ages 50+.

### Statistical methods

Descriptive statistics were used to describe the characteristics of women who attended screening at BreastScreen WA. Crude rescreening rates refer to unadjusted rates and are not age-standardized. Chi-square tests were used to compare screening characteristics for excluded versus included screening events. All analyses were stratified by age group and screening round. Mixed effect logistic regression models were used to investigate univariable and multivariable associations of breast density notification status with rescreening status, adjusted for other screening characteristics. Individual ID was included as a random effect. Interactions between breast density notification status and each of the screening characteristics were examined for evidence of effect modification. For the subset analysis, mixed effect logistic regression models were also used except the outcome was whether a woman rescreened at ages 50+. Statistical analyses were carried out in R (V4.0.2).

## Results

Table 1 shows characteristics of women who attended mammographic screening at BreastScreen WA between 2008 and 2017. The majority of screening events were from women in the targeted age range (78.4%), screening for the third/subsequent time (74.7%), who do not identify as Aboriginal (98.7%), with no FH of breast cancer (79.2%) or disability (98.4%), who speak English at home (86.0%), born in Australasia (60.8%), live in a major city (75.5%) and who have a high SEIFA (54.7%).

**Table 1:** Characteristics of women who attended mammographic screening at BreastScreen Western Australia, 2008–2017

Screening characteristic	Characteristic category	Total number of women *N* = 311,656^a^	Total number of screens *N* = 981,705 (% of screens)	Total number of screens notified *N* = 137,322 (% notified)
Age group	Younger age	75,078	131,288 (13.4%)	33,429 (25.5%)
	Targeted age	244,899	769,577 (78.4%)	98,125 (12.8%)
	Older age	40,029	80,840 (8.2%)	5768 (7.1%)
Screen Round	First	130,157	130,157 (13.3%)	27,654 (21.2%)
	Second	118,614	118,614 (12.1%)	21,758 (18.3%)
	Third+	223,439	732,934 (74.7%)	87,910 (12.0%)
Aboriginal	Yes	5479	12,918 (1.3%)	886 (6.9%)
	No	306,177	968,787 (98.7%)	136,436 (14.1%)
Disability	Yes	4661	15,235 (1.6%)	1337 (8.8%)
	No	306,995	966,470 (98.4%)	135,985 (14.1%)
Family history	Significant family history	22,396	103,456 (10.5%)	14,849 (14.4%)
	Family history	30,994	100,642 (10.3%)	14,079 (14%)
	No history	258,266	777,607 (79.2%)	108,394 (13.9%)
English spoken at home	Yes	267,917	844,067 (86.0%)	113,594 (13.5%)
	No	43,739	137,638 (14.0%)	23,728 (17.2%)
Country of birth	Australasia	190,638	596,694 (60.8%)	79,473 (13.3%)
	America/EU/UK	81,382	264,362 (26.9%)	32,389 (12.3%)
	Asia	29,022	89,891 (9.2%)	20,546 (22.9%)
	Africa	10,614	30,758 (3.1%)	4914 (16.0%)
ARIA^b^	Major cities	236,674	740,866 (75.5%)	107,975 (14.6%)
	Inner regional	45,510	120,196 (12.2%)	14,556 (12.1%)
	Outer regional	28,400	82,130 (8.4%)	9529 (11.6%)
	Remote	11,134	28,654 (2.9%)	3987 (13.9%)
	Very remote	4629	9859 (1.0%)	1275 (12.9%)
SEIFA^c^	Low	81,682	192,067 (19.6%)	24,296 (12.6%)
	Medium	104,124	252,643 (25.7%)	31,097 (12.3%)
	High	174,933	536,995 (54.7%)	81,929 (15.3%)

^a^Women attend screening multiple times and may have different characteristics for each screen. Therefore, totals within a category may not sum to the overall total number of women

^b^Accessibility/remoteness index of Australia

^c^Socio-economic indexes for areas

The proportion of screening events where women were notified they had dense breasts were higher for women aged 40–49 (25.5%), screened for the first time (21.2%), not Aboriginal (14.1%), with no disability (14.1%), with a significant FH (14.4%), who do not speak English at home (17.2%), born in Asia (22.9%), who live in major cities (14.6%) and have a higher SEIFA (15.3%).

Most of the screening characteristics of women excluded from the analysis were similar to those included in the study, but there was evidence that even small differences were statistically different (all *P* < 0.001; data not shown). Larger differences were seen for age group and screening round, where a larger proportion of younger women (19.2% vs. 13.4%) and first screeners (30.3% vs. 13.3%) were in the excluded versus included group, respectively.

**Fig. 2:**
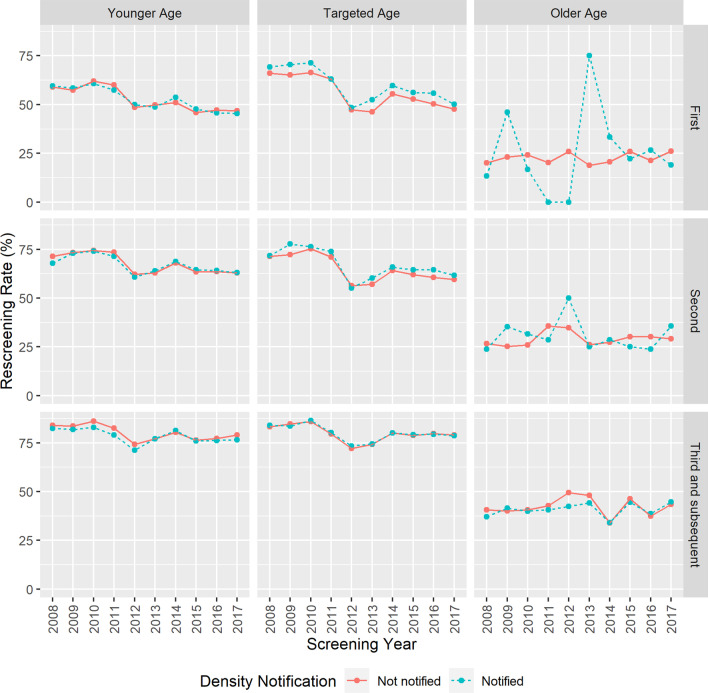
Crude rescreening rates by breast density notification status over time stratified by age group and screening round, BreastScreen Western Australia 2008–2017. Density notification identified by red: not notified and blue: notified

Figure 2 shows rescreening rates by breast density notification status over time (2008–2017) stratified by age group and screening round. Rescreening rates were similar for both notified and not notified women over time, with a couple of exceptions. Rescreening rates for notified women in the targeted age range screening for the first time appeared higher than those for not-notified women. The rescreening rates for notified older women screened in the first or second round were inconsistent, likely due to very small numbers of women in these groups, particularly notified women (see Table 2).

**Table 2:** Rescreening rates by breast density notification status and the corresponding odds ratios (OR) and 95% confidence intervals (CI) stratified by age group and screening round

Age	Proportion of screening events relating to women notified as dense who rescreened	Proportion of screening events relating to women not notified who rescreened	Unadjusted OR (95% CI)	Multivariable adjusted^a^ OR (95% CI)
*First screeners*				
Younger age	7657/15,032 (50.9%)	21,629/40,986 (52.8%)	0.93 (0.90–0.96)	0.89 (0.86–0.93)
Targeted age	7220/12,498 (57.8%)	33,569/59,866 (56.1%)	1.07 (1.03–1.11)	1.04 (1.01–1.09)
Older age	29/124 (23.4%)	372/1651 (22.5%)	1.05 (0.68–1.62)	0.99 (0.64–1.54)
*Second screeners*				
Younger age	5964/8993 (66.3%)	17,801/26,277 (67.7%)	0.94 (0.89, 0.99)	0.91 (0.87, 0.96)
Targeted age	8313/12,600 (66.0%)	44,470/68,720 (64.7%)	1.05 (1.01–1.09)	1.03 (0.99–1.07)
Older age	50/165 (30.3%)	530/1859 (28.5%)	1.09 (0.77–1.54)	1.07 (0.75–1.52)
*Third and subsequent screeners*				
Younger age	7325/9404 (77.9%)	24,492/30,596 (80.0%)	0.88 (0.82, 0.93)	0.84 (0.79, 0.90)
Targeted age	58,022/73,027 (79.5%)	430,847/542,866 (79.4%)	0.99 (0.96–1.01)	0.98 (0.95–1.00)
Older age	2273/5479 (41.5%)	29,567/71,562 (41.3%)	0.97 (0.91–1.05)	0.99 (0.92–1.07)

^a^The multivariable logistic regression model includes breast density notification status, family history, disability status, Aboriginal status, country of birth, English is spoken at home (yes/no), ARIA and SEIFA

### Associations between breast density notification and rescreening

**Fig. 3:**
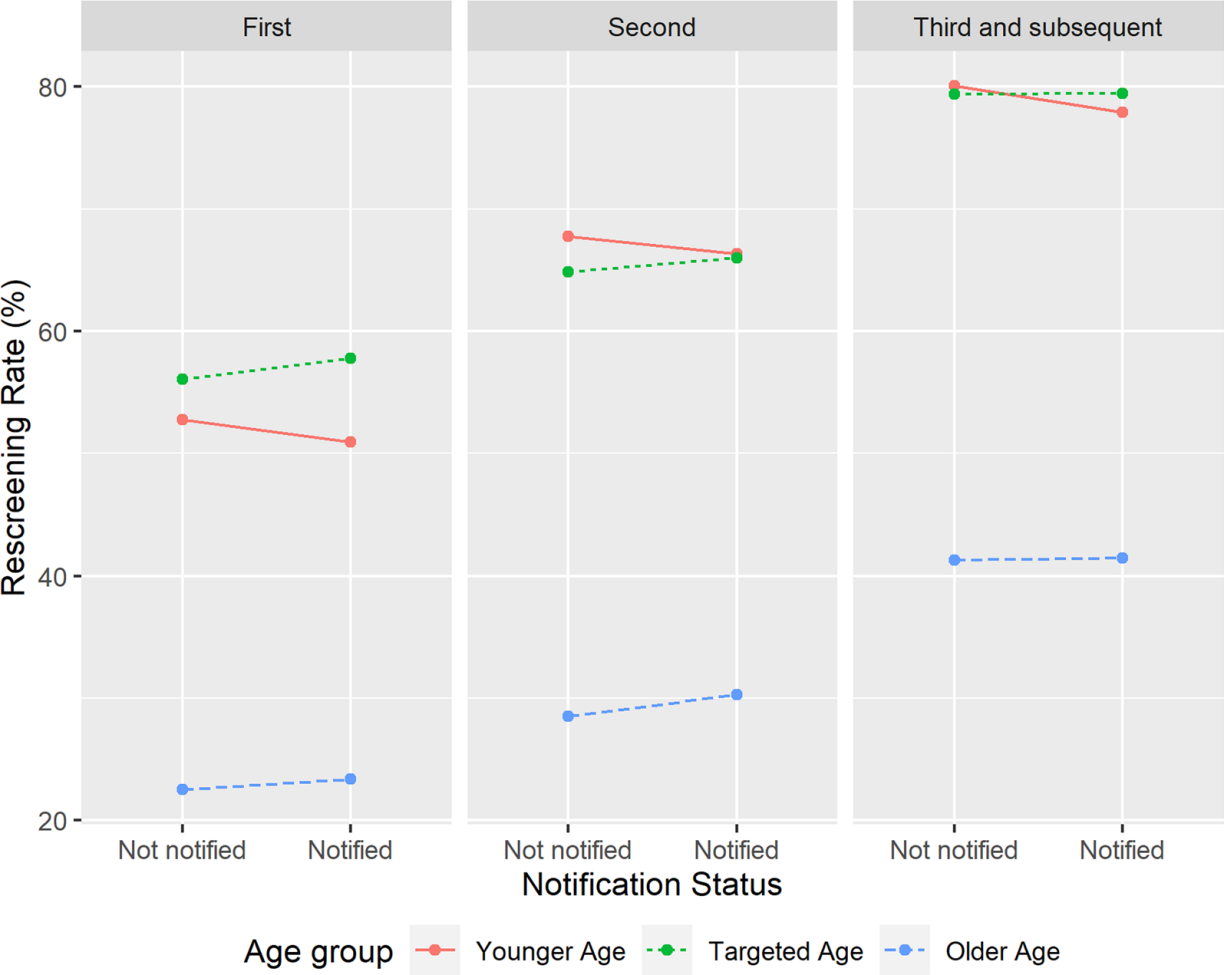
Crude rescreening rates by breast density notification status stratified by age group and screening round, BreastScreen Western Australia 2008–2017. Age group identified by red: younger age; green: targeted age and blue: older age

Table 2 shows crude rescreening rates by notification status and the corresponding odds ratios (OR) from the multivariable regression models. Tests for interaction (between breast density notification and other screening characteristics) showed evidence of effect modification by age group (*P*_interaction_ = 0.002) and screening round (*P*_interaction_ = 0.005). Women in the targeted age group were more likely to rescreen if notified they had dense breasts in the first round (compared to not-notified women; OR = 1.04, 95%CI 1.01–1.09, *P* = 0.02), but there was marginal evidence that notified women were less likely to rescreen if they were notified in the third+ round (compared to not-notified women; OR = 0.98, 95%CI 0.95–1.00, *P* = 0.048). However, the difference in the crude rescreening rates between those notified (58,022/73,027; 79.5%) and not-notified (430,847/542,866; 79.4%) was 0.1%, highlighting that the large sample sizes provide high statistical power to detect very small differences (the crude rate was actually higher in notified women, but the OR indicates an inverse association when Individual ID was fitted as a random effect in the regression models). Younger women who were notified they have dense breasts were less likely to return to screening, regardless of screening round (first: OR = 0.89, 95%CI 0.86–0.93; second: OR = 0.91, 95%CI 0.87–0.96; third/subsequent: OR = 0.84, 95%CI 0.79–0.90.). The corresponding differences in the crude rescreening rates between those notified and not notified were 1.9%, 1.4% and 2.1%. There was no evidence of association between breast density notification and the likelihood of rescreening in older women. Visualization of these associations are shown in Fig. 3.

There was moderate evidence of interactions of breast density notification status with FH (*P* < 0.001) and SEIFA (*P* = 0.003). Crude rescreening rates by breast density notification status and the corresponding odds ratios stratified by age group, screening round and either FH or SEIFA can be found in Additional file 1. Overall, the main message was largely unchanged with a few exceptions. From Additional file 1: Table S1(a), notified women in the targeted age range with a significant FH screening for the third/subsequent time were *more* likely to rescreen (compared to not-notified women; OR = 1.09, 95%CI 1.01–1.17). From Additional file 1: Table S2(a), notified women in the targeted age range with a low SEIFA screening for the second and third/subsequent time were also more likely to rescreen (compared to not-notified women; OR = 1.15, 95%CI 1.04–1.27; OR = 1.08, 95%CI 1.03–1.14 for second and third+ rounds, respectively). The results from Table 2 appear to be largely driven by women with a high SEIFA (Additional file 1: Table S2(c)).

### Investigation of whether notifying women if they have dense breasts at ages 40–49 is associated with the likelihood of returning to screening at ages 50+

Of the 46,420 screening events where younger women did not rescreen within the recommended screening interval, around 29% returned to screening at ages 50+ (Table 3). Of these, women who were notified they had dense breasts were less likely to return to screening when 50+ compared to women who were not-notified (OR = 0.52, 95%CI 0.48–0.55). Greater age increased the likelihood of these women returning to screening when 50+ (OR = 1.47, 95%CI 1.45–1.49), as did having a FH (FH: OR = 1.17, 95%CI 1.07–1.28; significant FH: OR = 1.13 95%CI 1.01–1.26) and being born in America/EU/UK (OR = 1.21, 95%CI 1.13–1.29; compared to Australasia). Women in this subset who lived in inner regional areas were more likely to return to screening when 50+ compared to those living in major cities (OR = 1.33, 95%CI 1.22–1.45), but less likely to return if they lived remotely (OR = 0.65, 95%CI 0.57–0.76) or very remotely (OR = 0.64 95%CI 0.51–0.79). Aboriginal women in this subset were less likely to return to screening at ages 50+ (OR = 0.78, 95%CI 0.66–0.93) as were women with low SEIFA (OR = 0.80, 95%CI 0.74–0.87, compared to high SEIFA).

**Table 3:** Multivariable odds ratios (OR) and 95% confidence intervals (CI) estimating the associations between rescreening status at ages 50+ (yes/no) and screening characteristics in a subset including only screening events from younger women (aged 40–49) who did not rescreen within the recommended screening interval (*N* = 46,420)

Screening characteristics	Characteristic category	Proportion of events where a woman returned to screening after 49 (%)	Multivariable adjusted odds ratio (95% CI)	*P* value^d^
Breast density notification	Not notified	10,804/33,937 (31.8%)	Reference	< 0.0001
	Notified	2495/12,483 (20.0%)	0.52 (0.48, 0.55)	
Age (years)	(continuous)		1.47 (1.45, 1.49)	< 0.0001
Screening round	First	5728/26,732 (21.4%)	Reference	< 0.0001
	Second	3760/11,505 (32.7%)	1.26 (1.19, 1.34)	
	Third+	3811/8183 (46.6%)	1.49 (1.39, 1.60)	
Aboriginal	No	12,966/44,775 (29.0%)	Reference	0.0062
	Yes	333/1645 (20.2%)	0.78 (0.66, 0.93)	
Family history (FH)	No history	10,995/38,997 (28.2%)	Reference	< 0.0001
	Family history	1468/4657 (31.5%)	1.17 (1.07, 1.28)	
	Significant FH	836/2766 (30.2%)	1.13 (1.01, 1.26)	
English at home	Yes	11,562/39,953 (28.9%)	Reference	0.5817
	No	1737/6467 (26.9%)	0.97 (0.88, 1.07)	
Disability	No	13,221/46,169 (28.6%)	Reference	0.5642
	Yes	78/251 (31.0%)	0.90 (0.63, 1.28)	
COB^a^	Australasia	8207/29,769 (27.6%)	Reference	< 0.0001
	America/EU/UK	3311/9822 (33.7%)	1.21 (1.13, 1.29)	
	Asia	1180/4451 (26.5%)	1.02 (0.90, 1.14)	
	Africa	601/2378 (25.3%)	0.90 (0.79, 1.02)	
SEIFA^b^	High	7938/27,291 (29.1%)	Reference	< 0.0001
	Medium	3237/10,666 (30.3%)	0.98 (0.91, 1.04)	
	Low	2124/8463 (25.1%)	0.80 (0.74, 0.87)	
ARIA^c^	Major city	10,117/34,988 (28.9%)	Reference	< 0.0001
	Inner regional	1732/5150 (33.6%)	1.33 (1.22, 1.45)	
	Outer regional	836/3164 (26.4%)	0.92 (0.83, 1.03)	
	Remote	427/2107 (20.3%)	0.65 (0.57, 0.76)	
	Very remote	187/1011 (18.5%)	0.64 (0.51, 0.79)	

^**a**^Country of birth

^b^Socio-Economic Indexes for Areas

^c^Accessibility/Remoteness Index of Australia

^d^− 2 log likelihood ratio test

## Discussion

With nearly 1 million screening events from over 300,000 women, this study shows that breast density notification does not deter women in the targeted age range from rescreening. Breast density notification does however appear to decrease the likelihood of rescreening for younger women. We also found that notified women aged 40–49 who do not rescreen within the recommended screening interval are also less likely to return to screening at ages when they are actively targeted (compared to non-notified women in the same subset).

Rescreening is one of the key performance indicators within the nationally accredited BreastScreen programs within Australia. In the absence of evidence-based screening recommendations specific for women with dense breasts, mammography is still best practice and high rescreening rates within women with dense breasts are therefore critical to promote early detection and better breast cancer outcomes. As BreastScreen WA is the only state in Australia that currently notifies women if they have dense breasts, it was important to determine that notification is not associated with reduced rescreening rates for women in the targeted age range. There is evidence that breast density notification may even increase the likelihood of rescreening in women in the targeted age range screening for the first time, a particularly worrisome group with the lowest reported rescreening rates according to the most recent BreastScreen Monitoring Report [5]. The crude rescreening rate for notified women in this group was 57.8% versus 56.1% for not notified women. The clinical relevance of a 1.7% improvement is dependent on the size and scope of the screening program but as all state-programs operate and report under the auspices of a large national funded program, these estimates are readily translatable across Australia and potentially other large publicly funded, population-based screening programs.

For younger women, the differences in the crude rescreening rates were 1.9%, 1.4% and 2.1% for those screened in the first, second, and third+ rounds, respectively. Again, the clinical relevance of these reductions in rescreening is dependent on the size and scope of the screening program. Without evidence-based screening recommendations for women with dense breasts, it is challenging to inform women with dense breasts about what action to take. The message appears to be particularly crucial for women aged 40–49 who are evidently deterred from BreastScreen WA, even later in life. Currently, breast density notification, imbedded in the routine mammography report, informs women that the sensitivity of mammography is reduced in women with dense breasts and recommends they consult their doctor. Australia has a two-tiered healthcare system. Private mammographic screening (and supplemental imaging) is also available to women of all ages, at their own cost (rebates are available with referral). Previous work indicates that around 50% of notified women (who attended BreastScreen WA) consult their doctor post-screening mammography and of those, ~ 50% are referred for further supplemental imaging. Of those, only 20% of notified women self-reported having an ultrasound due to their breast density [6]. Anecdotally, it is possible that younger women with dense breasts trust/prefer ultrasound over mammography for screening. As BreastScreen WA does not offer screening ultrasound, it is probable that younger women attend private providers that typically offer both mammography and ultrasound screening on the same day. It should be noted that BreastScreen programs are required to maintain national accreditation standards whilst private providers are not.

The results of this study suggest that perhaps the breast density notification information provided to women, particularly younger women, be re-evaluated to improve rescreening rates. However, whilst mammography is free for women aged 40–49 attending BreastScreen, national funding supports mammographic screening for women in the targeted age range only. Younger women are not actively invited to participate in screening (not until age 50) and therefore, improving rescreening rates within younger women is not necessarily a priority within the state BreastScreen programs.

This may have important implications for participation and rescreening rates within BreastScreen WA when women reach the targeted age range. Our results show that young women notified they have dense breasts who do not rescreen in their 40’s are much less likely to return to BreastScreen WA when invited at age 50 (compared to those who were not notified). Once in the private system, these women may prefer to stay with their private provider once they turn 50, possibly contributing to the 56% national participation rate in the BreastScreen programs. From Table 3, other predictors explaining why younger women do not return to BreastScreen WA when in the targeted age range may relate to access of care like ARIA, SEIFA and Aboriginal status. Despite mobile mammography units that travel to rural and remote areas of Western Australia every 2 years, access to screening (and follow-up assessment/treatment if required) is still challenging in a state that is comparable, in land-size, to Western Europe. Increasing legislation in the USA, mandating breast density notification, has substantially increased the number of studies assessing the impact of notification on screening outcomes but most studies are small in size or compared overall summaries of screening outcomes before and after legislation enactment, instead of using individual-level data [10]. This study is the first of its kind, using individual-level data to estimate the association between breast density notification and rescreening within a population-based mammographic screening program. Other strengths include its size and duration, with 981,705 screening events from over 311,656 women conducted over 12 years.

A limitation of this study is that its large sample size means very small differences are statistically significant but not necessarily clinically relevant. For (another) example, the significant difference in the crude rescreening rates between notified and not-notified targeted women screening for the third+ round was 0.1%, which we argue is not clinically significant. Another potential limitation was that the excluded screening events were more likely to be younger women and/or attending screening for the first time due to higher recall rates in these women. First round screeners are recalled to assessment around 10% versus 4% for subsequent round screeners and younger women screen for the first time than older women. Screening events that resulted in recall were excluded as they do not receive the routine results letter containing the dense breast notification. Women with P.O boxes were also excluded as they are not mapped to socio-demographic data by the ABS. Finally, although BreastScreen WA is the largest and only publicly funded screening service in the State, women can access screening through private providers and we do not have data from private providers.

## Conclusion

In summary, this is the largest investigation of the association between breast density notification and rescreening within a population-based mammographic screening program and includes over 12 years of individual-level data. We showed that breast density notification is unlikely to deter women from rescreening, except perhaps for younger women. The results will directly inform BreastScreen programs in Australia as well as other population-based screening providers outside Australia who notify women about breast density or are considering implementing breast density notification. Further research is critically needed to determine screening recommendations for women with dense breasts. In the meantime, we need ways to inform women, particularly younger women, about their breast density in a way that reinforces the importance of continued mammographic rescreening, vital for early detection and better breast cancer outcomes.

## Supplementary Information

**Additional file 1** Associations between rescreening rates and breast density notification status further stratified by familiy history and SEIFA.

## Data Availability

The datasets generated and analysed during the current study are not publicly available but can be made available upon request (and pending approval) from BreastScreen Western Australia.
