# Supplementary material for: The impact of breast density notification on rescreening rates within a population-based mammographic screening program
Source: Breast Cancer Res. 2022 Jan 15;24:5. doi: 10.1186/s13058-021-01499-4 (PMC8760641; doi:10.1186/s13058-021-01499-4)
Supplement: Supplementary file 1 — Additional file 1 Associations between rescreening rates and breast density notification status further stratified by familiy history and SEIFA. [file 13058_2021_1499_MOESM1_ESM.docx]

**Additional file 1: Table S1: Rescreening rates by breast density notification status and the corresponding odds ratios (OR) and 95% Confidence Intervals (CI) stratified by age group, screening round and family history - (a) significant family history, (b) family history, (c) no family history**

| 1. **Significant family history** | |  |  |  |
| --- | --- | --- | --- | --- |
| Age Group | Proportion of screening events relating to women **notified as dense** who rescreened | Proportion of screening events relating to women **not notified** who rescreened | Unadjusted OR (95% CI) | Multivariable adjusted* OR (95% CI) |
|  | First screeners | | | |
| Younger age | 803/1155 (69.5%) | 2154/3063 (70.3%) | 0.96 (0.83-1.12) | 0.92 (0.79-1.06) |
| Targeted age | 474/652 (72.7%) | 2200/3070 (71.7%) | 1.05 (0.87-1.23) | 0.99 (0.82-1.20) |
| Older age | 5/16 (31.2%) | 68/170 (40.0%) | 0.68 (0.23-2.05) | 0.63 (0.20-1.98) |
|  | Second screeners | | | |
| Younger age | 706/884 (79.9%) | 1977/2505 (78.9%) | 1.06 (0.88-1.28) | 1.02 (0.84-1.24) |
| Targeted age | 477/740 (64.5%) | 2428/3891 (62.4) | 1.09 (0.93-1.29) | 1.09 (0.92-1.29) |
| Older age | 8/18 (44.4%) | 89/193 (46.1%) | 0.93 (0.35-2.47) | 0.73 (0.27-2.01) |
|  | Third and subsequent screeners | | | |
| Younger age | 1793/2041 (89.0%) | 5404/5982 (90.3%) | 0.85 (0.71-1.02) | 0.83 (0.69-0.99) |
| Targeted age | 6759/8594 (78.6%) | 46,529/60,222 (77.3%) | 1.11 (1.03-1.20) | **1.09 (1.01-1.17)** |
| Older age | 479/776 (61.7%) | 5588/9511 (58.8%) | 1.10 (0.91-1.32) | 1.10 (0.91-1.32) |
| **(b) Family history** | Proportion of screening events relating to women **notified as dense** who rescreened | Proportion of screening events relating to women **not notified** who rescreened | Unadjusted OR (95% CI) | Multivariable adjusted* OR (95% CI) |
|  | First screeners | | | |
| Younger age | 744/1431 (52.0%) | 2199/3950 (55.7%) | 0.86 (0.76-0.97) | **0.85 (0.75-0.96)** |
| Targeted age | 625/1052 (59.4%) | 2932/4894 (59.9%) | 0.98 (0.86-1.12) | 0.97 (0.84-1.11) |
| Older age | 4/12 (33.3%) | 49/208 (23.6%) | 1.62 (0.47-5.62) | 1.77 (0.46-6.80) |
|  | Second screeners | | | |
| Younger age | 638/948 (67.3%) | 1899/2767 (68.6%) | 0.94 (0.80-1.10) | 0.92 (0.78-1.08) |
| Targeted age | 770/1154 (66.7%) | 4056/6072 (66.8%) | 1.00 (0.87-1.14) | 0.98 (0.86-1.12) |
| Older age | 7/22 (31.8%) | 74/248 (29.8%) | 1.10 (0.43-2.80) | 1.10 (0.42-2.89) |
|  | Third and subsequent screeners | | | |
| Younger age | 807/1037 (77.8%) | 2705/3516 (76.9%) | 1.05 (0.89-1.25) | 1.06 (0.89-1.26) |
| Targeted age | 6236/7778 (80.2%) | 45,137/56,089 (80.5%) | 0.97 (0.91-1.04) | 0.96 (0.90-1.03) |
| Older age | 278/645 (43.1%) | 3917/8819 (44.4%) | 0.90 (0.73-1.10) | 0.92 (0.75-1.13) |
| **(c) No family history** | Proportion of screening events relating to women **notified as dense** who rescreened | Proportion of screening events relating to women **not notified** who rescreened | Unadjusted OR (95% CI) | Multivariable adjusted* OR (95% CI) |
|  | First screeners | | | |
| Younger age | 6110/12,446 (49.1%) | 17,276/33,973 (50.9%) | 0.93 (0.89-0.97) | **0.90 (0.86-0.93)** |
| Targeted age | 6121/10,794 (56.7%) | 28,437/51,902 (54.8%) | 1.08 (1.04-1.13) | **1.06 (1.02-1.11)** |
| Older age | 20/96 (20.8%) | 255/1273 (20.0%) | 1.05 (0.63-1.75) | 1.01 (0.60-1.67) |
|  | Second screeners | | | |
| Younger age | 4620/7161 (64.5%) | 13,925/21,005 (66.3%) | 0.92 (0.87-0.98) | **0.90 (0.85-0.96)** |
| Targeted age | 7066/10,706 (66.0%) | 38,086/58,757 (64.8%) | 1.05 (1.01-1.10) | 1.03 (0.99-1.08) |
| Older age | 35/125 (28.0%) | 367/1418 (25.9%) | 1.11 (0.74-1.67) | 1.07 (0.71-1.62) |
|  | Third and subsequent screeners | | | |
| Younger age | 4725/6353 (74.4%) | 16,383/21,098 (77.7%) | 0.84 (0.78-0.90) | **0.81 (0.76-0.87)** |
| Targeted age | 45,027/56,655 (79.5%) | 339181/426555 (79.5%) | 0.98 (0.96-1.01) | **0.97 (0.94-0.99)** |
| Older age | 1516/4058 (37.4%) | 20,062/53,232 (37.7%) | 0.96 (0.88-1.05) | 0.99 (0.91-1.08) |

| **(a) Low SEIFA** |  |  |  |  |
| --- | --- | --- | --- | --- |
| Age Group | Proportion of screening events relating to women **notified as dense** who rescreened | Proportion of screening events relating to women **not notified** who rescreened | Unadjusted OR (95% CI) | Multivariable adjusted* OR (95% CI) |
|  | First screeners | | | |
| Younger age | 1179/2417 (48.9%) | 3781/7505 (50.4%) | 0.94 (0.86-1.03) | **0.88 (0.80-0.96)** |
| Targeted age | 1210/2276 (53.2%) | 6364/11,831 (53.8%) | 0.98 (0.89-1.07) | 0.95 (0.87-1.04) |
| Older age | 9/23 (39.1%) | 60/315 (19.0%) | 2.73 (1.13-6.61) | 2.48 (0.97-6.32) |
|  | Second screeners | | | |
| Younger age | 925/1395 (66.3%) | 3076/4615 (66.7%) | 0.98 (0.87-1.12) | 0.92 (0.81-1.05) |
| Targeted age | 1492/2235 (66.8%) | 8406/13,373 (62.9%) | 1.19 (1.08-1.30) | **1.15 (1.04-1.27)** |
| Older age | 9/19 (47.4%) | 94/364 (25.8%) | 2.56 (1.02-6.56) | 2.45 (0.95-6.37) |
|  | Third and subsequent screeners | | | |
| Younger age | 1140/1489 (76.6%) | 4122/5265 (78.3%) | 0.90 (0.78-1.05) | **0.85 (0.73-0.99)** |
| Targeted age | 10,451/13,187 (79.3%) | 83,524/107,617 (77.6%) | 1.10 (1.05-1.16) | **1.08 (1.03-1.14)** |
| Older age | 566/1255 (45.1%) | 7307/16,886 (43.3%) | 1.08 (0.94-1.25) | 1.10 (0.95-1.27) |

**Additional file 1: Table S2: Rescreening rates by breast density notification status and the corresponding odds ratios (OR) and 95% Confidence Intervals (CI) stratified by age group, screening round and SEIFA - (a) Low SEIFA, (b) Medium SEIFA, (c) High SEIFA**

| **(b) Medium SEIFA** | Proportion of screening events relating to women **notified as dense** who rescreened | Proportion of screening events relating to women **not notified** who rescreened | Unadjusted OR (95% CI) | Multivariable adjusted* OR (95% CI) |
| --- | --- | --- | --- | --- |
|  | First screeners | | | |
| Younger age | 1649/3254 (50.7%) | 5004/9594 (52.2%) | 0.94 (0.87-1.02) | **0.90 (0.83-0.97)** |
| Targeted age | 1693/2949 (57.4%) | 8684/15,755 (55.1%) | 1.10 (1.01-1.19) | **1.08 (1.00-1.17)** |
| Older age | 8/26 (30.8%) | 84/382 (22.0%) | 1.58 (0.66-3.75) | 1.43 (0.58-3.54) |
|  | Second screeners | | | |
| Younger age | 1274/1926 (66.1%) | 4090/6055 (67.5%) | 0.94 (0.84-1.05) | 0.90 (0.81-1.01) |
| Targeted age | 1957/2970 (65.9%) | 11,690/18,142 (64.4%) | 1.07 (0.98-1.16) | 1.05 (0.96-1.14) |
| Older age | 6/25 (24.0%) | 124/463 (26.8%) | 0.86 (0.34-2.21) | 0.85 (0.32-2.22) |
|  | Third and subsequent screeners | | | |
| Younger age | 1507/1926 (78.2%) | 5709/7144 (79.9%) | 0.91 (0.80-1.04) | **0.88 (0.77-1.01)** |
| Targeted age | 13,134/16,649 (78.9%) | 113580/143804 (79.0%) | 0.98 (0.94-1.03) | 0.97 (0.93-1.02) |
| Older age | 603/1372 (44.0%) | 8533/20,207 (42.2%) | 1.08 (0.94-1.24) | 1.10 (0.96-1.26) |
| **(c) High SEIFA** | Proportion of screening events relating to women **notified as dense** who rescreened | Proportion of screening events relating to women **not notified** who rescreened | Unadjusted OR (95% CI) | Multivariable adjusted* OR (95% CI) |
|  | First screeners | | | |
| Younger age | 4829/9361 (51.6%) | 12,844/23,887 (53.8%) | 0.92 (0.87-0.96) | **0.90 (0.85-0.94)** |
| Targeted age | 4317/7273 (59.4%) | 18,521/32,280 (57.4%) | 1.08 (1.03-1.14) | **1.07 (1.02-1.13)** |
| Older age | 12/75 (16.0%) | 228/954 (23.9%) | 0.61 (0.32-1.14) | 0.57 (0.30-1.10) |
|  | Second screeners | | | |
| Younger age | 3765/5672 (66.4%) | 10,635/15,607 (68.1%) | 0.92 (0.87-0.98) | **0.91 (0.85-0.97**) |
| Targeted age | 4864/7395 (65.8%) | 24,474/37,205 (65.8%) | 1.00 (0.95-1.05) | 0.99 (0.94-1.04) |
| Older age | 35/121 (28.9%) | 312/1032 (30.2%) | 0.94 (0.62-1.42) | 0.99 (0.65-1.50) |
|  | Third and subsequent screeners | | | |
| Younger age | 4678/5989 (78.1%) | 14,661/18,187 (80.6%) | 0.85 (0.79-0.92) | **0.83 (0.76-0.89)** |
| Targeted age | 34,437/43,191 (79.7%) | 233743/291445 (80.2%) | 0.96 (0.93-0.99) | **0.95 (0.92-0.98)** |
| Older age | 1104/2852 (38.7%) | 13,727/34,469 (39.8%) | 0.91 (0.82-1.00) | 0.92 (0.83-1.01) |

*The multivariable logistic regression model includes density notification status, family history, disability status, Aboriginal and Torres Strait Islander status, country of birth, English is spoken at home (yes/no), ARIA and SEIFA; Bold font indicates CI that does not include 1.
